# Prediagnosis Smoking Cessation and Overall Survival Among Patients With Non–Small Cell Lung Cancer

**DOI:** 10.1001/jamanetworkopen.2023.11966

**Published:** 2023-05-05

**Authors:** Xinan Wang, Christopher W. Romero-Gutierrez, Jui Kothari, Andrea Shafer, Yi Li, David C. Christiani

**Affiliations:** 1 Department of Environmental Health, Harvard T.H. Chan School of Public Health, Harvard University, Boston, Massachusetts; 2 Department of Biostatistics, School of Public Health, University of Michigan, Ann Arbor; 3 Pulmonary and Critical Care Division, Department of Medicine, Massachusetts General Hospital, Harvard Medical School, Boston

## Abstract

**Importance:**

Lung cancer remains the leading cause of cancer-related death globally; non–small cell lung cancer (NSCLC) accounts for 85% of all lung cancer cases, and cigarette smoking is the factor most significantly associated with its risk. However, little is known about the association of years since prediagnosis smoking cessation and cumulative smoking with overall survival (OS) following a lung cancer diagnosis.

**Objective:**

To characterize the association of years since smoking cessation before diagnosis and cumulative smoking pack-years with OS in patients with NSCLC in a lung cancer survivor cohort.

**Design, Setting, and Participants:**

The cohort study involved patients with NSCLC who were recruited to the Boston Lung Cancer Survival Cohort at Massachusetts General Hospital (Boston, Massachusetts) between 1992 and 2022. Patients’ smoking history and baseline clinicopathological characteristics were prospectively collected through questionnaires, and OS following lung cancer diagnosis was regularly updated.

**Exposures:**

Duration of smoking cessation before a lung cancer diagnosis.

**Main Outcomes and Measures:**

The primary outcome was the association of detailed smoking history with OS following a lung cancer diagnosis.

**Results:**

Of 5594 patients with NSCLC (mean [SD] age, 65.6 [10.8] years; 2987 men [53.4%]), 795 (14.2%) were never smokers, 3308 (59.1%) were former smokers, and 1491 (26.7%) were current smokers. Cox regression analysis suggested that former smokers had 26% higher mortality (hazard ratio [HR], 1.26; 95% CI, 1.13-1.40; *P* < .001) and current smokers had 68% higher mortality (HR, 1.68; 95% CI, 1.50-1.89; *P* < .001) compared with never smokers. Log_2_-transformed years since smoking cessation before diagnosis were associated with significantly lower mortality among ever smokers (HR, 0.96; 95% CI, 0.93-0.99; *P* = .003). Subgroup analysis, stratified by clinical stage at diagnosis, revealed that former and current smokers had even shorter OS among patients with early-stage disease.

**Conclusions and Relevance:**

In this cohort study of patients with NSCLC, quitting smoking early was associated with lower mortality following a lung cancer diagnosis, and the association of smoking history with OS may have varied depending on clinical stage at diagnosis, potentially owing to the differing treatment regimens and efficacy associated with smoking exposure following diagnosis. Detailed smoking history collection should be incorporated into future epidemiological and clinical studies to improve lung cancer prognosis and treatment selection.

## Key Points

**Question:**

Is prediagnosis smoking cessation associated with overall survival among patients with non–small cell lung cancer?

**Findings:**

In this long-term follow-up cohort study of lung cancer survivors, compared with never smokers, former and current smokers had 26% and 68% higher mortality, respectively, and log_2_-transformed year since smoking cessation before diagnosis was associated with significantly lower mortality among ever smokers.

**Meaning:**

These findings suggest that quitting smoking early is associated with lower mortality following a lung cancer diagnosis, and the association of smoking history with overall survival may vary depending on clinical stage at diagnosis, potentially because of the differing treatment regimens and efficacy associated with smoking exposure following diagnosis.

## Teaser

This cohort study examines the association of years since smoking cessation before diagnosis and cumulative smoking pack-years with overall survival in patients with non–small cell lung cancer.

## Introduction

It is estimated that more than half a million individuals in the US are living with lung and bronchus cancer, the largest portion among all those living with cancer.^1,2^ Non–small cell lung cancer (NSCLC) accounts for 85% of all lung cancer cases, and smoking (cigarette or tobacco) is the factor most significantly associated with it.^3^ Although lung cancer incidence has kept decreasing as a result of tobacco control, the prognosis and 5-year survival rates remain poor.^4,5,6^ The relative 5-year survival rate estimated from the Surveillance, Epidemiology, and End Results Program’s data is approximately 22.9%, well below rates for many other common cancers.^1^ In addition, the disease has the highest economic burden among all the cancers.^7^ Because current medical interventions have generated only mild to moderate improvements in survival among a small subset of patients with lung cancer, improving these approaches by more effectively targeting the behavioral changes of patients may help improve their prognosis.

Smoking activities may cause molecular changes, as recent evidence has revealed that smoking is associated with certain oncogenic driver mutations and tumor mutation burden (TMB), a metric quantifying the number of somatic mutations occurring within the cancer genome.^8^ For example, the number of mutations in genes that promote oncogenic activities increases with smoking pack-years, and smoking cessation can reduce TMB, thus lowering risks of cancer progression and mortality.^8,9^ Moreover, cigarette smoking was reported to be associated with increased systemic level of various inflammatory biomarkers, whereas smoking cessation, over a period, may be associated with lower levels of these biomarkers, demonstrating the benefit of quitting smoking.^10^

There is also evidence of systemic changes after smoking cessation. The literature of smoking cessation has focused on a variety of health outcomes after cessation. For example, several studies have found that smoking cessation earlier in life can contribute to improved longevity.^11^ A few studies^12,13,14^ have found that smoking cessation can improve outcomes in diabetes and blood pressure, and former smokers have lower lung cancer incidence after quitting.^15^ Even though these studies have largely helped understand the life-cycle effects of smoking, few of them have explored the granular associations of time since smoking cessation and cumulative smoking pack-years with overall survival (OS) following a lung cancer diagnosis in a long-term follow-up setting.^9,16,17,18^ The aforementioned molecular changes, coupled with the association reported in Sheikh et al^9^ between smoking cessation after diagnosis and progression-free survival and OS in patients with early-stage lung cancer, may indicate an advantage of smoking cessation on cancer survival. Therefore, the association of prediagnosis smoking cessation, smoking intensity, and cumulative smoking with lung cancer survival outcomes merits further study. In this study, we assessed the granular associations of years since smoking cessation, cigarettes per day, and smoking pack-years with OS following a lung cancer diagnosis and explored the heterogeneity of outcomes across various clinical stages at diagnosis, on the basis of a prospective long-term cohort of patients with NSCLC recruited in Boston, Massachusetts, between 1992 and 2022.

## Methods

### Study Sample and Design

This cohort study was approved by the institutional review board of Massachusetts General Hospital. Patients who provided written informed consent were included. This study followed the Strengthening the Reporting of Observational Studies in Epidemiology (STROBE) reporting guideline.

Our cohort consisted of patients who received a diagnosis of NSCLC at Massachusetts General Hospital. Information on clinical stage (stage I, IA, IB, II, IIA, IIB, III, IIIA, IIIB, or IV) was recorded at the time of diagnosis and was further categorized into early-stage (I-IIIA) and late-stage (IIIB-IV) disease. In addition, patients’ lung cancer histologic profiles were obtained from medical records with the following classifications: lung adenocarcinoma, squamous cell carcinoma, other NSCLC including large cell lung carcinoma, adenocarcinoma in situ (formerly bronchoalveolar carcinoma), and unspecified NSCLC.

At enrollment, hard-copy questionnaires collected baseline information on patient age, sex, self-reported race (Asian, Black, White, or other racial group, including American Indian or Alaska Native, Native Hawaiian or Pacific Islander, and multiracial), ethnicity (Hispanic or non-Hispanic), education (grade school, high school, high school graduate, vocational or technical school after high school, college or associate’s degrees, college graduate, graduate or professional school, and other), family history of lung cancer (yes or no), and smoking-related information. Data on race and ethnicity were collected because they reflect the distribution of the survival cohort, and we were interested in whether they were associated with survival outcomes. Missing covariate information was minimal (<3% for most covariates) (Table 1).

**Table 1.** Baseline Characteristics of Study Population According to Smoking Status at Diagnosis

Characteristic	Patients with NSCLC (N = 5594)			*P* value^a^
	Never smokers (n = 795)	Former smokers (n = 3308)	Current smokers (n = 1491)	
Age, mean (SD), y	62.4 (13.3)	68.4 (9.55)	61.7 (9.98)	<.001
Education level				
College graduate	168 (21.1)	457 (13.8)	117 (7.8)	<.001
Graduate or professional school	228 (28.7)	371 (11.2)	68 (4.6)	
High school graduate	88 (11.1)	673 (20.3)	352 (23.6)	
Some college or associates degree	150 (18.9)	715 (21.6)	287 (19.2)	
Some grade school	27 (3.4)	181 (5.5)	135 (9.1)	
Some high school	39 (4.9)	414 (12.5)	276 (18.5)	
Vocational or technical school	28 (3.5)	114 (3.4)	79 (5.3)	
Other	5 (0.6)	32 (1.0)	15 (1.0)	
Missing	62 (7.8)	351 (10.6)	162 (10.9)	
Self-reported race				
Asian	79 (10)	37 (1.1)	11 (0.8)	<.001
Black	19 (2.4)	42 (1.3)	40 (2.8)	
White	671 (84.4)	3153 (95.3)	1397 (93.7)	
Other^b^	23 (2.9)	86 (2.6)	1 (0.1)	
Missing	12 (1.5)	34 (1.0)	19 (1.3)	
Sex				
Female	271 (34.1)	1617 (48.9)	703 (47.1)	<.001
Male	519 (65.3)	1682 (50.8)	786 (52.7)	
Missing	5 (0.6)	9 (0.3)	2 (0.1)	
Self-reported ethnicity				
Hispanic	21 (2.6)	26 (0.8)	21 (1.4)	<.001
Non-Hispanic	695 (87.4)	2935 (88.7)	1295 (86.9)	
Missing	79 (9.9)	347 (10.5)	175 (11.7)	
Lung cancer histologic profile				
Adenocarcinoma	616 (77.5)	2056 (62.2)	786 (52.7)	<.001
Squamous cell carcinoma	44 (5.5)	705 (21.3)	385 (25.8)	
NSCLC not specified	58 (7.3)	228 (6.9)	152 (10.2)	
Other NSCLC	77 (9.7)	319 (9.6)	168 (11.3)	
Clinical stage at diagnosis				
IA-IIIA	444 (55.8)	2279 (68.9)	948 (63.6)	<.001
IIIB-IV	345 (43.4)	997 (30.1)	535 (35.9)	
Missing	6 (0.8)	32 (1.0)	8 (0.5)	
Family history of lung cancer				
No	171 (21.5)	583 (17.6)	119 (8.0)	<.001
Yes	186 (23.4)	955 (28.9)	434 (29.1)	
Missing	438 (55.1)	1770 (53.5)	938 (62.9)	
Smoking characteristics, mean (SD)				
Pack-years, No.	NA	44.3 (32.1)	57.0 (32.9)	<.001
Cigarettes per day, No.	NA	24.4 (14.1)	24.9 (12.7)	.06
Age started smoking, y	NA	17.2 (4.2)	16.7 (5.2)	<.001
Age stopped smoking, y	NA	51.1 (13.5)	60.1 (10.7)	<.001
Years of smoking cessation, No.	NA	17.3 (13)	1.7 (6.3)	<.001

Abbreviations: NA, not applicable; NSCLC, non–small cell lung cancer.

^a^ *P* values were obtained from Kruskal-Wallis tests for continuous variables and from χ^2^ tests for categorical variables.

^b^ Includes American Indian or Alaska Native, Native Hawaiian or Pacific Islander, and multiracial.

The exposures of interest were smoking status (never, former, or current), cigarettes per day, smoking pack-years, and time lag (in years) between smoking cessation and diagnosis. Smoking status was assessed at the baseline questionnaire. For patients who were classified as former and current smokers, information was collected on cigarettes per day, smoking start date, and smoking stop date for former smokers. Pack-years for current smokers was calculated using cigarettes per day, age the participant started smoking, and age at the time of death or age at censoring. For former smokers, pack-years was calculated using cigarettes per day and number of years when the patient continued smoking. Forty-eight patients who missed the last contact date were excluded from the analyses with OS.

### Follow-up and Data Collection

Patients were followed-up regularly every 12 to 18 months. To capture the events of death, we used hospital clinical and cancer registry data. When data were incomplete, we conducted web searches of participants in obituaries or websites with links to the National Death Index and assessed whether patients had been pronounced dead at the time of follow-up, as well as their date of death.

### Statistical Analysis

To compare baseline characteristics between the 3 smoking status categories, Kruskal-Wallis tests and χ^2^ tests were used for continuous and categorical variables, respectively. OS was from the date of lung cancer diagnosis to death, or the last contact documented in the data, whichever came first. Survival distributions were estimated using the Kaplan-Meier estimator, and log-rank tests were used to test for survival differences across different groups. Cox proportional hazards models were fitted to obtain the estimates of hazard ratios (HRs) in univariable and multivariable models. Variables with a marginal association with *P* < .05 were included in the multivariable model. Base 2 log transformation was used for years since smoking cessation, cigarettes per day, and smoking pack-years to meet the linearity assumption and to facilitate easy interpretation.^8,16^ All *P* values are 2-sided, confidence intervals are set to be at the 95% level, and significance is predefined to be at *P* < .05.

## Results

### Study Population and Baseline Characteristics

The cohort was composed of 5594 patients with NSCLC (mean [SD] age, 65.6 [10.8] years; 2987 men [53.4%]), including 795 (14.2%) never smokers, 3308 (59.1%) former smokers, and 1491 (26.7%) current smokers (eFigure in Supplement 1 and Table 1). Our cohort was predominantly White (5221 patients [93.3%]) and non-Hispanic (4925 patients [88.0%]). Current smokers had a significantly higher number of smoking pack-years (mean [SD], 57.0 [32.9] pack-years) compared with former smokers (mean [SD], 44.3 [32.1] pack-years). Former smokers tended to receive their diagnosis later in life; the mean (SD) age at diagnosis for former smokers was 68.4 (9.55) years, compared with 62.4 (13.3) years for never smokers and 61.7 (9.98) years for current smokers. In addition, a higher proportion of never smokers attained a college degree or higher (396 patients [49.8%]) compared with former (828 patients [25.0%]) and current (185 patients [12.4%]) smokers. Of these patients, 3842 (69.1%) died over the course of follow-up, including 1177 (79.3%) current smokers, 2191 (66.8%) former smokers, and 474 (59.6%) never smokers.

We observed much heterogeneity in cancer characteristics (Table 1). Lung adenocarcinomas represented 3458 diagnoses (61.8%), and there was heterogeneity in cancer types across various smoking groups; notably, former and current smokers had significantly higher rates of squamous cell carcinoma (705 patients [21.3%] and 385 patients [25.8%], respectively) compared with never smokers (44 patients [5.5%]) (Table 1). A larger proportion of former and current smokers had their diagnosis at clinical stage I to IIIA (2279 patients [68.9%] and 948 patients [63.6%], respectively) compared with never smokers (444 patients [55.8%]). Less than one-third of all patients (1575 patients [28.2%]) had a family history of lung cancer, but only 2448 (43.8%) provided this information.

### Association of Prediagnosis Smoking History With OS

Smoking status was significantly associated with OS outcomes, with the longest median OS of 58.9 months (95% CI, 51.9-67.4 months) among never smokers, followed by former smokers (median OS, 51.2 months; 95% CI, 47.7-54.6 months) and current smokers (median OS, 34.0 months; 95% CI, 29.1-42.3 months) (Figure, panel A). In crude analysis, former smokers (HR, 1.19; 95% CI, 1.08-1.31; *P* < .001) and current smokers (HR, 1.43; 95% CI, 1.28-1.59; *P* < .001) had an increased risk of death following lung cancer diagnosis compared with never smokers (eTable 1 in Supplement 1). Doubling smoking pack-years was associated with shorter OS (HR, 1.07; 95% CI, 1.05-1.08; *P* < .001). In addition, female sex, younger age, early clinical stage at diagnosis, and lung adenocarcinoma were significantly associated with prolonged OS. To control for potential confounders, we adjusted for covariates that met statistical significance (*P* < .05), including age, sex, histologic profile, and clinical stage (eTable 2 in Supplement 1), and former smokers continued to have 26% higher mortality (HR, 1.26; 95% CI, 1.13-1.40; *P* < .001) and current smokers had 68% higher mortality (HR, 1.68; 95% CI, 1.50-1.89; *P* < .001) compared with never smokers (Table 2).

**Figure.**
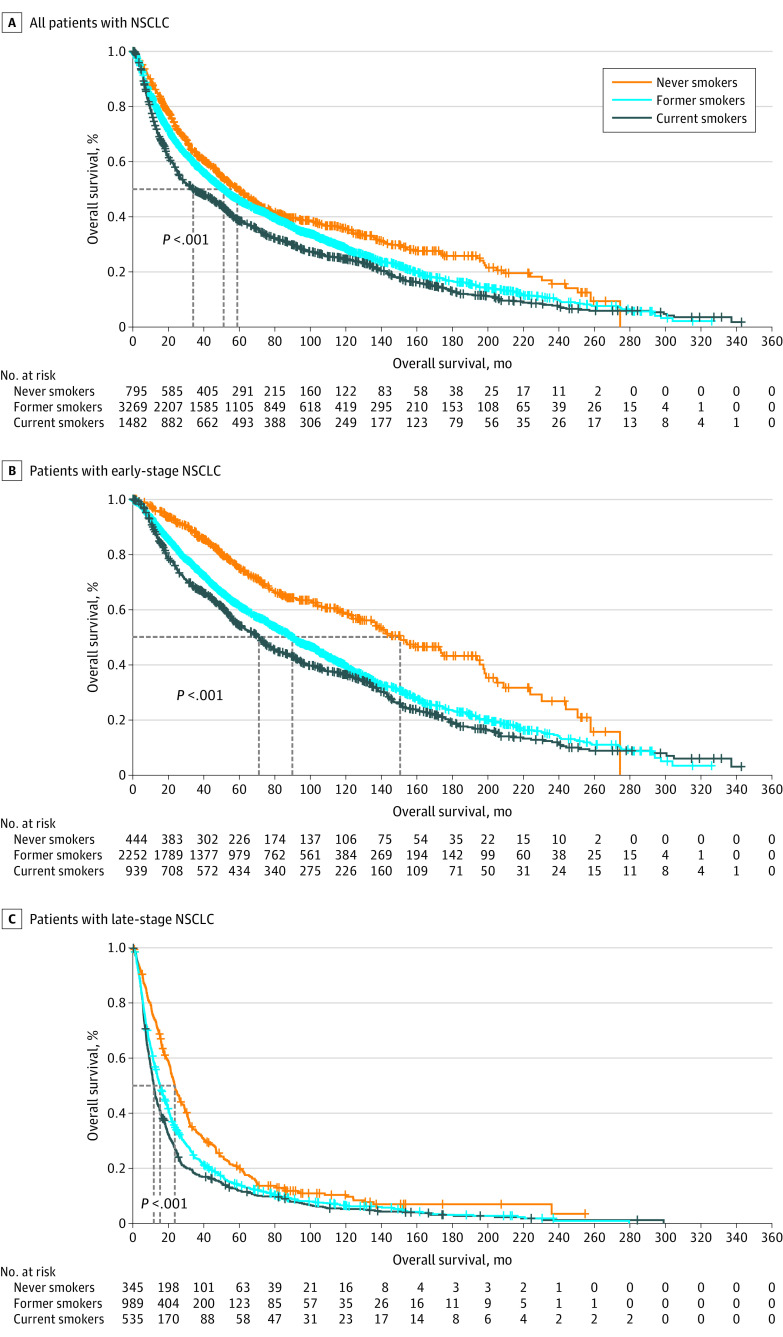
Overall Survival Among Patients With Non–Small Cell Lung Cancer (NSCLC) by Smoking Status at Diagnosis Graphs show survival among all patients with NSCLC (A), patients with early-stage NSCLC (B), and patients with late-stage NSCLC (C).

**Table 2.** Multivariable Analysis of Smoking Status at Diagnosis for Overall Survival Following Lung Cancer Diagnosis

Covariates^a^	HR (95% CI)	*P* value
Smoking status		
Never	1 [Reference]	NA
Former	1.26 (1.13-1.40)	<.001
Current	1.68 (1.50-1.89)	<.001
Sex		
Male	1 [Reference]	<.001
Female	0.72 (0.68-0.78)	
Age at diagnosis (per 1-y increase)	1.02 (1.02-1.03)	<.001
Lung cancer histologic profile		
Lung adenocarcinoma	1 [Reference]	NA
Lung squamous cell carcinoma	1.31 (1.20-1.42)	<.001
Other non–small cell lung cancer vs lung adenocarcinoma	0.83 (0.75-0.92)	<.001
Clinical stage at diagnosis		
I-IIIA	1 [Reference]	<.001
IIIB-IV	4.28 (3.96-4.63)	

Abbreviations: HR, hazard ratio; NA, not applicable.

^a^ Covariates that met the statistical significance were included in the multivariable analyses.

In subgroup analysis stratified by clinical stage at diagnosis, history of smoking was associated with a shorter OS in patients with early-stage disease (former smoker vs never smoker HR, 1.37; 95% CI, 1.16-1.63; *P* < .001; current smoker vs never smoker HR, 1.93; 95% CI, 1.61-2.32; *P* < .001) (Figure, panel B and eTable 3 in Supplement 1) compared with patients who received a diagnosis of stage IIIB to IV disease (former smoker vs never smoker HR, 1.19; 95% CI, 1.03-1.36; *P* = .01; current smoker vs never smoker HR, 1.43; 95% CI, 1.23-1.66; *P* < .001) (Figure, panel C, and eTable 4 in Supplement 1) after controlling for potential confounders.

### Association of Detailed Smoking History With OS in Ever Smokers

In addition to the categorical smoking statuses, we investigated the association of detailed smoking history with OS among patients who ever smoked. In ever smokers, doubling smoking pack-years was significantly associated with shorter OS (HR, 1.07; 95% CI, 1.04-1.11; *P* < .001) (Table 3). Furthermore, an increased HR was observed in patients who received a diagnosis of stage I to IIIA disease (HR, 1.09; 95% CI, 1.06-1.12; *P* < .001) compared with patients with late-stage disease (HR, 1.06; 95% CI, 1.03-1.08; *P* < .001) (eTables 5 and 6 in Supplement 1). In addition, log_2_-transformed cigarettes per day was also associated with a reduced OS in ever smoker patients after controlling for the same covariates (HR, 1.08; 95% CI, 1.03-1.13; *P* < .001) (eTable 7 in Supplement 1). For former smokers, doubling years since smoking cessation was associated with prolonged OS (HR, 0.96; 95% CI, 0.93-1.00; *P* = .04) (eTable 8 in Supplement 1).

**Table 3.** Association of Smoking Pack-Years With Overall Survival in Ever Smokers

Covariates	HR (95% CI)	*P* value
Doubling smoking pack-years	1.07 (1.04-1.11)	<.001
Sex		
Male	1 [Reference]	<.001
Female	0.75 (0.70-0.82)	
Age at diagnosis (per 1-y increase)	1.02 (1.01-1.02)	<.001
Lung cancer histologic profile		
Lung adenocarcinoma	1 [Reference]	NA
Lung squamous cell carcinoma	1.28 (1.17-1.41)	<.001
Other non–small cell lung cancer	0.80 (0.71-0.89)	<.001
Clinical stage at diagnosis		
I-IIIA	1 [Reference]	<.001
IIIB-IV	4.20 (3.84-4.60)	

Abbreviations: HR, hazard ratio; NA, not applicable.

Finally, we included both smoking pack-years and years from commencement of smoking cessation to diagnosis in the model. Doubling years of smoking cessation was associated with prolonged survival (HR, 0.96; 95% CI, 0.93-0.99; *P* = .003) (Table 4), whereas the HR for death increased along with increasing number of smoking pack-years, but the result was not significant (HR, 1.03; 95% CI, 0.98-1.07; *P* = .21), likely because of the heterogeneity of clinical stage at diagnosis. There was no association of log_2_-transformed cigarettes per day with OS (HR, 1.04; 95% CI, 0.99-1.10; *P* = .14) (eTable 9 in Supplement 1). In both stratified analyses of early-stage and late-stage diagnoses, doubling the years of smoking cessation was associated with prolonged OS after adjusting for other covariates (early-stage HR, 0.95; 95% CI, 0.91-0.98; *P* = .005; late-stage HR, 0.95; 95% CI, 0.91-1.00; *P* = .04) (eTables 10 and 11 in Supplement 1). In contrast, doubling smoking pack-years was not significantly associated with a shorter OS in patients with early-stage disease (HR, 1.06; 95% CI, 1.00-1.12; *P* = .05) or in patients with late-stage disease (HR, 0.98; 95% CI, 0.92-1.05; *P* = .57).

**Table 4.** Association of Smoking Pack-Years With Years Since Smoking Cessation and Overall Survival in Ever Smokers

Covariates	HR (95% CI)	*P* value
Doubling smoking pack-years	1.03 (0.98-1.07)	.21
Doubling years since smoking cessation	0.96 (0.93-0.99)	.003
Sex		
Male	1 [Reference]	<.001
Female	0.75 (0.69-0.83)	
Age at diagnosis (per 1-y increase)	1.03 (1.02-1.03)	<.001
Lung cancer histologic profile		
Lung adenocarcinoma	1 [Reference]	NA
Lung squamous cell carcinoma	1.25 (1.12-1.39)	<.001
Other non–small cell lung cancer	0.76 (0.66-0.86)	<.001
Clinical stage at diagnosis		
I-IIIA	1 [Reference]	<.001
IIIB-IV	4.23 (3.81-4.70)	

Abbreviations: HR, hazard ratio; NA, not applicable.

## Discussion

The findings of this cohort study indicate that both former and current smokers have higher mortality compared with never smokers following a NSCLC diagnosis. Former smokers experienced an 26% increased mortality (HR, 1.26; 95% CI, 1.13-1.40), whereas current smokers experienced a 68% increased mortality (HR, 1.68; 95% CI, 1.50-1.89) compared with never smokers. Another study^19^ found the 5-year survival rate following a lung adenocarcinoma diagnosis for current smokers was 16% compared with 23% for never smokers, which is in agreement with our modeling results. In addition, doubling the years of smoking cessation preceding a lung cancer diagnosis was significantly associated with improved OS, and this association was modified by the clinical stage at diagnosis, possibly owing to differing treatment regimens and efficacy according to smoking exposure following diagnosis. In agreement with the literature,^20^ we found that never smokers had a higher proportion of diagnoses at later stages, as well as a higher proportion of lung adenocarcinomas, compared with former and current smokers.

Our large prospective cohort helped us detect the profound benefits of smoking cessation, which is associated with reduced mortality among former smokers, compared with current smokers. Other studies^11,21^ have shown that former smokers have a reduction in their risk of myocardial infarction and stroke, as well as improved longevity, compared with current smokers. In line with the 2020 Surgeon General’s Report^22^ demonstrating that quitting smoking even after a cancer diagnosis is associated with improved cancer treatment outcomes, our findings suggest that the benefit of smoking cessation persists even after lung cancer is diagnosed. Our results may also resonate with the literature,^10,11,23^ which has demonstrated that cells, and potentially the entire human body, undergo substantial changes after smoking cessation and the precise mechanism merits further exploration.

This study does not stratify by the cause of death, and there might be various plausible explanations for our results, ranging from direct reduced probability of death from lung cancer as well as from other causes, such as a reduction of risk from cardiovascular disease or other types of cancers. For example, smoking cessation has been associated with increases in the circulation of endothelial progenitor cells that can increase angiogenesis, improving tissue repair.^24,25^ This could contribute to improved survival from cardiovascular diseases in former smokers. However, there is also evidence to suggest that our results are largely due to reductions in deaths from lung cancer. Somatic mutation burden also tends to change after exposure of tobacco smoke ceases, suggesting that smoking cessation could ameliorate the carcinogenic effect of tobacco exposure over time.^26,27^ As a result, lung cancer cases among former smokers who quit smoking a long time before their diagnosis may be more analogous to cases in never smokers, who experience better survival rates than current smokers.^27^

The observed heterogeneity of smoking status and cumulative smoking exposure among patients who receive a diagnosis at different clinical stages is important because they may inform biomarker discovery and patient selection for emerging therapeutics. For example, the potential benefits of increased smoking pack-years in later stages of lung cancer also indicate that certain immunogenomic features might be leading to different treatment selections. Patients with late-stage disease are more likely to receive systemic treatment including targeted treatment and immune checkpoint inhibitors. The positive association of increased smoking pack-years with TMB in advanced NSCLC has been reported and, thus, leads to an improved clinical outcome compared with never smokers.^23,28^

### Strengths and Limitations

Our study has several strengths. First, our cohort featured patients with detailed smoking histories, allowing us to investigate the survival trajectory of current and former smokers compared with never smokers following a lung cancer diagnosis. In contrast, much of the available literature focuses on the comparison between current and never smokers. Second, detailed smoking intensity and history, including cigarettes per day, smoking pack-years, and smoking cessation in years, were well-documented in our study, which allowed more granular characterization of various smoking trajectories before and after diagnosis. The wide range of smoking cessation periods in former smokers, ranging from a few years to several decades, enabled us to investigate the benefits of smoking cessation more precisely and gave us confidence in the results for long-term smoking cessation. Finally, our study was conducted on the basis of a 30-year follow-up cohort that is still growing, and it provided a unique opportunity to explore the association with long-term survival across all clinical stages and in various lung cancer histologic profiles.

This study also has several limitations. First, our cohort lacks racial diversity, since more than 90% of patients in the study were White, and the wide 95% CIs for race effect estimates suggest these effects are not sufficiently powered. This may limit the generalizability of our results to non-Hispanic White patients. Therefore, additional research is needed in larger and more diverse cohorts. Second, we did not control for potential lifestyle confounders. It is plausible that former smokers experienced lifestyle changes following smoking cessation, and it could be lifestyle changes that contributed to the improved survival. For example, several studies found that a subset of former smokers increased their physical activity and developed other healthy lifestyle habits.^25,26^ Third, we did not include detailed treatment information and, hence, could not differentiate treatment-related outcomes from smoking cessation–related outcomes. Fourth, recall bias in smoking history is possible because of the retrospective nature of smoking-related information. Fifth, our study did not differentiate by cause of death, leading to the possibility that the improved survival was due to a reduction of other morbidities, rather than a reduction in lung cancer–specific mortality.^29,30^

## Conclusions

In this cohort of patients with NSCLC, quitting smoking early in life was associated with reduced mortality following a lung cancer diagnosis. The association of smoking history with OS may have varied depending on clinical stage at diagnosis, potentially owing to the differing treatment regimens and efficacy associated with smoking exposure following diagnosis. To better inform treatment selection and to improve lung cancer prognosis, detailed smoking history should be collected and studied further in conjunction with molecular and genomic biomarkers in future epidemiological and clinical studies.

## Notes

Supplement 1. **eFigure.** Patient Enrollment Flowchart **eTable 1.** Univariable Analysis for Overall Survival Following Lung Cancer Diagnosis **eTable 2.** Heterogeneous Effect of Smoking Status by Clinical Stage on Overall Survival Following Lung Cancer Diagnosis **eTable 3.** Multivariable Analysis of Smoking Status at Diagnosis for Overall Survival Following Lung Cancer Diagnosis in Early-Stage NSCLC Patients **eTable 4.** Multivariable Analysis of Smoking Status at Diagnosis for Overall Survival Following Lung Cancer Diagnosis in Late-Stage NSCLC Patients **eTable 5.** Effect of Smoking Pack-year on Overall Survival in Early-Stage Ever Smoker NSCLC Patients **eTable 6.** Effect of Smoking Pack-year on Overall Survival in Late-Stage Ever Smoker NSCLC Patients **eTable 7.** Effect of Cigarettes per Day and Years Since Smoking Cessation on Overall Survival in Ever Smoker NSCLC Patients **eTable 8.** Association Between Years Since Smoking Cessation and Overall Survival in Former Smokers **eTable 9.** Effect of Cigarettes per Day and Years Since Smoking Cessation on Overall Survival in Ever Smoker NSCLC Patients **eTable 10.** Effect of Smoking Pack-years and Years Since Smoking Cessation on Overall Survival in Early-Stage Ever Smoker NSCLC Patients **eTable 11.** Effect of Smoking Pack-years and Years Since Smoking Cessation on Overall Survival in Late-Stage Ever Smoker NSCLC Patients

Supplement 2.
Data Sharing Statement
