# Supplementary material for: Prediagnosis Smoking Cessation and Overall Survival Among Patients With Non–Small Cell Lung Cancer
Source: JAMA Netw Open. 2023 May 5;6(5):e2311966. doi: 10.1001/jamanetworkopen.2023.11966 (PMC10163381; doi:10.1001/jamanetworkopen.2023.11966)
Supplement: Supplement 1. — eFigure. Patient Enrollment Flowchart eTable 1. Univariable Analysis for Overall Survival Following Lung Cancer Diagnosis eTable 2. Heterogeneous Effect of Smoking Status by Clinical Stage on Overall Survival Following Lung Cancer Diagnosis eTable 3. Multivariable Analysis of Smoking Status at Diagnosis for Overall Survival Following Lung Cancer Diagnosis in Early-Stage NSCLC Patients eTable 4. Multivariable Analysis of Smoking Status at Diagnosis for Overall Survival Following Lung Cancer Diagnosis in Late-Stage NSCLC Patients eTable 5. Effect of Smoking Pack-year on Overall Survival in Early-Stage Ever Smoker NSCLC Patients eTable 6. Effect of Smoking Pack-year on Overall Survival in Late-Stage Ever Smoker NSCLC Patients eTable 7. Effect of Cigarettes per Day and Years Since Smoking Cessation on Overall Survival in Ever Smoker NSCLC Patients eTable 8. Association Between Years Since Smoking Cessation and Overall Survival in Former Smokers eTable 9. Effect of Cigarettes per Day and Years Since Smoking Cessation on Overall Survival in Ever Smoker NSCLC Patients eTable 10. Effect of Smoking Pack-years and Years Since Smoking Cessation on Overall Survival in Early-Stage Ever Smoker NSCLC Patients eTable 11. Effect of Smoking Pack-years and Years Since Smoking Cessation on Overall Survival in Late-Stage Ever Smoker NSCLC Patients [file jamanetwopen-e2311966-s001.pdf]

Supplemental Online Content

Wang X, Romero-Gutierrez CW, Kothari J, Shafer A, Li Y, Christiani DC. Prediagnosis smoking cessation and overall survival among patients with non–small cell lung cancer. *JAMA Netw Open*. 2023;6(5):e2311966. doi:10.1001/jamanetworkopen.2023.11966

eFigure. Patient Enrollment Flowchart
eTable 1. Univariable Analysis for Overall Survival Following Lung Cancer Diagnosis
eTable 2. Heterogeneous Effect of Smoking Status by Clinical Stage on Overall Survival Following Lung Cancer Diagnosis
eTable 3. Multivariable Analysis of Smoking Status at Diagnosis for Overall Survival Following Lung Cancer Diagnosis in Early-Stage NSCLC Patients
eTable 4. Multivariable Analysis of Smoking Status at Diagnosis for Overall Survival Following Lung Cancer Diagnosis in Late-Stage NSCLC Patients
eTable 5. Effect of Smoking Pack-year on Overall Survival in Early-Stage Ever Smoker NSCLC Patients
eTable 6. Effect of Smoking Pack-year on Overall Survival in Late-Stage Ever Smoker NSCLC Patients
eTable 7. Effect of Cigarettes per Day and Years Since Smoking Cessation on Overall Survival in Ever Smoker NSCLC Patients
eTable 8. Association Between Years Since Smoking Cessation and Overall Survival in Former Smokers
eTable 9. Effect of Cigarettes per Day and Years Since Smoking Cessation on Overall Survival in Ever Smoker NSCLC Patients
eTable 10. Effect of Smoking Pack-years and Years Since Smoking Cessation on Overall Survival in Early-Stage Ever Smoker NSCLC Patients
eTable 11. Effect of Smoking Pack-years and Years Since Smoking Cessation on Overall Survival in Late-Stage Ever Smoker NSCLC Patients

This supplemental material has been provided by the authors to give readers additional information about their work.

© 2023 Wang X et al. *JAMA Network Open.*

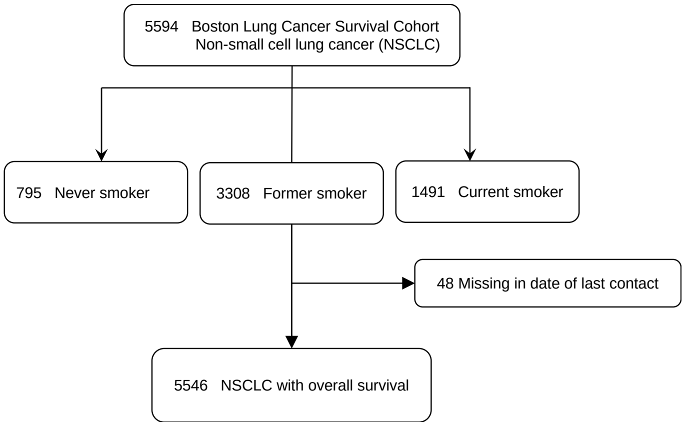

eFigure. Patient Enrollment Flowchart

The study was conducted on 5594 patients with a diagnosis of non-small cell lung cancer. Patients with date of last contact were included in the analysis with overall survival.

eTable 1. Univariable Analysis for Overall Survival Following Lung Cancer Diagnosis

Covariates	HR (95% CI)	P-value
Smoking status		
Never	1 [Reference]	
Former	1.19 (1.08-1.31)	< 0.001
Current	1.43 (1.28-1.59)	< 0.001
Doubling smoking pack-years	1.07 (1.05-1.08)	< 0.001
Doubling time since smoking cessation	1.00 (0.98-1.03)	0.82
Sex		
Male	1 [Reference]	
Female	0.70 (0.66-0.75)	< 0.001
Age at diagnosis	1.01 (1.01 - 1.02)	< 0.001
Race		
Asian	0.84 (0.67-1.07)	0.16
Black	1.00 (0.79-1.27)	0.99
White	1 [Reference]	
Other	1.03 (0.78-1.37)	0.83
Lung cancer histology		
Lung adenocarcinoma	1 [Reference]	
Lung squamous cell carcinoma	1.32 (1.22-1.43)	< 0.001
Other NSCLC	0.93 (0.84-1.03)	0.18
Unspecified	1.10 (0.97-1.24)	0.13
Clinical stage		
I-IIIA	1 [Reference]	
IIIB-IV	3.68 (3.44-3.93)	< 0.001

© 2023 Wang X et al. *JAMA Network Open.*

eTable 2. Heterogeneous Effect of Smoking Status by Clinical Stage on Overall Survival Following Lung Cancer Diagnosis

Covariates	HR (95% CI)	P-value
Smoking status		
Never	1 [Reference]	
Former	1.36 (1.15-1.61)	< 0.001
Current	1.81 (1.51-2.16)	< 0.001
Clinical stage		
I-IIIA	1 [Reference]	
IIIB-IV	4.87 (3.98-5.95)	< 0.001
Sex		
Male	1 [Reference]	
Female	0.74 (0.69-0.80)	< 0.001
Age at diagnosis (per 1-year increase)	1.02 (1.02-1.03)	< 0.001
Lung cancer histology		
Lung adenocarcinoma	1 [Reference]	
Lung squamous cell carcinoma	1.25 (1.15-1.36)	< 0.001
Other NSCLC	0.81 (0.73-0.90)	< 0.001
Interaction Term		
Never smoker * stage I-IIIA	1 [Reference]	
Former smoker * stage IIIB-IV	0.81 (0.65-1.01)	0.06
Current smoker * stage IIIB-IV	0.81 (0.64-1.02)	0.08

eTable 3. Multivariable Analysis of Smoking Status at Diagnosis for Overall Survival Following Lung Cancer Diagnosis in Early-Stage NSCLC Patients

Covariates	HR (95% CI)	P-value
Smoking status		
Never	1 [Reference]	
Former	1.33 (1.12-1.58)	< 0.001
Current	1.90 (1.59-2.27)	< 0.001
Sex		
Male	1 [Reference]	
Female	0.74 (0.67-0.81)	< 0.001
Age at diagnosis (per 1-year increase)	1.04 (1.03-1.04)	< 0.001
Lung cancer histology		
Lung adenocarcinoma	1 [Reference]	
Lung squamous cell carcinoma	1.39 (1.25-1.54)	< 0.001
Other NSCLC	0.92 (0.81-1.06)	0.24

eTable 4. Multivariable Analysis of Smoking Status at Diagnosis for Overall Survival Following Lung Cancer Diagnosis in Late-Stage NSCLC Patients

Covariates	HR (95% CI)	P-value
Smoking status		
Never	1 [Reference]	
Former	1.24 (1.07-1.43)	0.004
Current	1.56 (1.33-1.82)	< 0.001
Sex		
Male	1 [Reference]	
Female	0.76 (0.68-0.84)	< 0.001
Age at diagnosis (per 1-year increase)	1.01 (1.01-1.02)	< 0.001
Lung cancer histology		
Lung adenocarcinoma	1 [Reference]	
Lung squamous cell carcinoma	1.03 (0.89-1.19)	0.69
Other NSCLC	0.77 (0.65-0.90)	0.001

© 2023 Wang X et al. *JAMA Network Open.*

eTable 5. Effect of Smoking Pack-year on Overall Survival in Early-Stage Ever Smoker NSCLC Patients

Covariates	HR (95% CI)	P-value
Doubling smoking pack-years	1.09 (1.06-1.12)	< 0.001
Sex		
Male	1 [Reference]	
Female	0.79 (0.72-0.88)	< 0.001
Age at diagnosis (per 1-year increase)	1.03 (1.03-1.04)	< 0.001
Lung cancer histology		
Lung adenocarcinoma	1 [Reference]	
Lung squamous cell carcinoma	1.35 (1.21-1.51)	< 0.001
Other NSCLC	0.90 (0.78-1.03)	0.13

eTable 6. Effect of Smoking Pack-year on Overall Survival in Late-Stage Ever Smoker NSCLC Patients

Covariates	HR (95% CI)	P-value
Doubling smoking pack-years	1.06 (1.03-1.08)	< 0.001
Sex		
Male	1 [Reference]	
Female	0.76 (0.68-0.85)	< 0.001
Age at diagnosis (per 1-year increase)	1.01 (1.00-1.01)	0.003
Lung cancer histology		
Lung adenocarcinoma	1 [Reference]	
Lung squamous cell carcinoma	1.02 (0.87-1.19)	0.84
Other NSCLC	0.72 (0.61-0.85)	< 0.001

eTable 7. Effect of Cigarettes per Day and Years Since Smoking Cessation on Overall Survival in Ever Smoker NSCLC Patients

Covariates	HR (95% CI)	P-value
Doubling cigarettes per day	1.08 (1.03-1.13)	< .001
Sex		
Male	1 [Reference]	
Female	0.76 (0.70-0.82)	< .001
Age at diagnosis (per 1-year increase)	1.02 (1.01-1.02)	< .001
Lung cancer histology		
Lung adenocarcinoma	1 [Reference]	
Lung squamous cell carcinoma	1.33 (1.21-1.45)	< .001
Other NSCLC	0.85 (0.76-0.94)	< .001
Clinical stage		
I-IIIA	1 [Reference]	
IIIB-IV	4.17 (3.83-4.54)	< .001

eTable 8. Association Between Years Since Smoking Cessation and Overall Survival in Former Smokers

Covariates	HR (95% CI)	P-value
Doubling years since smoking cessation	0.96 (0.93-1.00)	0.04
Sex		
Male	1 [Reference]	
Female	0.72 (0.65-0.79)	< .001
Age at diagnosis (per 1-year increase)	1.03 (1.02-1.03)	< .001
Lung cancer histology		
Lung adenocarcinoma	1 [Reference]	
Lung squamous cell carcinoma	1.28 (1.14-1.44)	< .001
Other NSCLC	0.74 (0.64-0.85)	< .001
Clinical stage		
I-IIIA	1 [Reference]	
IIIB-IV	4.12 (3.68-4.62)	< .001

© 2023 Wang X et al. *JAMA Network Open.*

eTable 9. Effect of Cigarettes per Day and Years Since Smoking Cessation on Overall Survival in Ever Smoker NSCLC Patients

Covariates	HR (95% CI)	P-value
Doubling cigarettes per day	1.04 (0.99-1.10)	0.14
Doubling years since smoking cessation	0.95 (0.92-0.97)	< .001
Sex		
Male	1 [Reference]	
Female	0.76 (0.69-0.83)	< .001
Age at diagnosis (per 1-year increase)	1.03 (1.02-1.03)	< .001
Lung cancer histology		
Lung adenocarcinoma	1 [Reference]	
Lung squamous cell carcinoma	1.25 (1.13-1.40)	< .001
Other NSCLC	0.77 (0.67-0.87)	< .001
Clinical stage		
I-IIIA	1 [Reference]	
IIIB-IV	4.15 (3.74-4.61)	< .001

eTable 10. Effect of Smoking Pack-years and Years Since Smoking Cessation on Overall Survival in Early-Stage Ever Smoker NSCLC Patients

Covariates	HR (95% CI)	P-value
Doubling smoking pack-years	1.06 (1.00-1.12)	0.05
Doubling years since smoking cessation	0.95 (0.91-0.98)	0.005
Sex		
Male	1 [Reference]	
Female	0.78 (0.69-0.87)	< 0.001
Age at diagnosis	1.04 (1.03-1.05)	< 0.001
Lung cancer histology		
Lung adenocarcinoma	1 [Reference]	
Lung squamous cell carcinoma	1.30 (1.14-1.48)	< 0.001
Other NSCLC	0.92 (0.78-1.09)	0.34

eTable 11. Effect of Smoking Pack-years and Years Since Smoking Cessation on Overall Survival in Late-Stage Ever Smoker NSCLC Patients

Covariates	HR (95% CI)	P-value
Doubling smoking pack-years	0.98 (0.92-1.05)	0.57
Doubling years since smoking cessation	0.95 (0.91-1.00)	0.04
Sex		
Male	1 [Reference]	
Female	0.79 (0.69-0.92)	0.002
Age at diagnosis (per 1-year increase)	1.01 (1.00-1.02)	0.002
Lung cancer histology		
Lung adenocarcinoma	1 [Reference]	
Lung squamous cell carcinoma	1.03 (0.85-1.25)	0.74
Other NSCLC	0.62 (0.50-0.76)	< 0.001

© 2023 Wang X et al. *JAMA Network Open.*
