# Supplementary material for: Prediagnosis Smoking Cessation and Overall Survival Among Patients With Non–Small Cell Lung Cancer
Source: JAMA Netw Open. 2023 May 5;6(5):e2311966. doi: 10.1001/jamanetworkopen.2023.11966 (PMC10163381; doi:10.1001/jamanetworkopen.2023.11966)
Supplement: Supplement 2. — Data Sharing Statement [file jamanetwopen-e2311966-s002.pdf]

Data Sharing Statement

Wang. Prediagnosis Smoking Cessation and Overall Survival Among Patients With Non-Small Cell Lung Cancer. *JAMA Netw Open*. Published May 05, 2023.
doi:10.1001/jamanetworkopen.2023.11966

Data
Data available: The data that support the finding of our study is available upon request from the corresponding author through a data use agreement (DUA) process.
